# A Data-Driven Clustering Method for Discovering Profiles in the Dynamics of Major Depressive Disorder Using a Smartphone-Based Ecological Momentary Assessment of Mood

**DOI:** 10.3389/fpsyt.2022.755809

**Published:** 2022-03-17

**Authors:** Claire R. van Genugten, Josien Schuurmans, Adriaan W. Hoogendoorn, Ricardo Araya, Gerhard Andersson, Rosa M. Baños, Thomas Berger, Cristina Botella, Arlinda Cerga Pashoja, Roman Cieslak, David D. Ebert, Azucena García-Palacios, Jean-Baptiste Hazo, Rocío Herrero, Jérôme Holtzmann, Lise Kemmeren, Annet Kleiboer, Tobias Krieger, Anna Rogala, Ingrid Titzler, Naira Topooco, Johannes H. Smit, Heleen Riper

**Affiliations:** ^1^Department of Psychiatry, Amsterdam Public Health Institute, Amsterdam UMC, Vrije Universiteit, Amsterdam, Netherlands; ^2^Department of Clinical, Neuro and Developmental Psychology, Faculty of Behavioural and Movement Sciences, Vrije Universiteit, Amsterdam, Netherlands; ^3^Institute of Psychiatry Psychology and Neurosciences, King's College London, London, United Kingdom; ^4^Department of Behavioural Sciences and Learning, Department of Biomedical and Clinical Sciences, Linköping University, Linköping, Sweden; ^5^Department of Clinical Neuroscience, Centre for Psychiatry Research, Karolinska Institutet, Stockholm, Sweden; ^6^Polibienestar Research Institute, University of Valencia, Valencia, Spain; ^7^CIBERObn Physiopathology of Obesity and Nutrition, Instituto de Salud Carlos III, Madrid, Spain; ^8^Department of Personality, Evaluation and Psychological Treatment, Faculty of Psychology, University of Valencia, Valencia, Spain; ^9^Department of Clinical Psychology, University of Bern, Bern, Switzerland; ^10^Department of Basic and Clinical Psychology and Psychobiology, Faculty of Health Sciences, Jaume I University, Castellon de la Plana, Spain; ^11^Department of Population Health, London School of Hygiene and Tropical Medicine, London, United Kingdom; ^12^Faculty of Psychology, SWPS University of Social Sciences and Humanities, Warsaw, Poland; ^13^Lyda Hill Institute for Human Resilience, Colorado Springs, CO, United States; ^14^Department for Sport and Health Sciences, Technical University (TU) Munich, Munich, Germany; ^15^Eceve, Unit 1123, Inserm, University of Paris, Health Economics Research Unit, Assistance Publique-Hôpitaux de Paris, Paris, France; ^16^Unité de Recherche en Economie de la Santé, Assistance Publique, Hôpitaux de Paris, Paris, France; ^17^Mood Disorders and Emotional Pathologies Unit, Centre Expert Depression Résistante Fondation Fondamental, Pôle de Psychiatrie, Neurologie et Rééducation Neurologique, University Hospital Grenoble Alpes, Grenoble, France; ^18^Department of Clinical Psychology and Psychotherapy, Institute of Psychology, Friedrich-Alexander-University Erlangen-Nürnberg, Erlangen, Germany; ^19^Center for m2Health, Palo Alto, CA, United States; ^20^Institute of Telepsychiatry, University of Southern Denmark, Odense, Denmark; ^21^University of Turku, Faculty of Medicine, Turku, Finland

**Keywords:** depression, ecological momentary assessment, mood dynamics, mood instability, heterogeneity, cluster analysis

## Abstract

**Background:**

Although major depressive disorder (MDD) is characterized by a pervasive negative mood, research indicates that the mood of depressed patients is rarely entirely stagnant. It is often dynamic, distinguished by highs and lows, and it is highly responsive to external and internal regulatory processes. Mood dynamics can be defined as a combination of mood variability (the magnitude of the mood changes) and emotional inertia (the speed of mood shifts). The purpose of this study is to explore various distinctive profiles in real-time monitored mood dynamics among MDD patients in routine mental healthcare.

**Methods:**

Ecological momentary assessment (EMA) data were collected as part of the cross-European E-COMPARED trial, in which approximately half of the patients were randomly assigned to receive the blended Cognitive Behavioral Therapy (bCBT). In this study a subsample of the bCBT group was included (*n* = 287). As part of bCBT, patients were prompted to rate their current mood (on a 1–10 scale) using a smartphone-based EMA application. During the first week of treatment, the patients were prompted to rate their mood on three separate occasions during the day. Latent profile analyses were subsequently applied to identify distinct profiles based on average mood, mood variability, and emotional inertia across the monitoring period.

**Results:**

Overall, four profiles were identified, which we labeled as: (1) “very negative and least variable mood” (*n* = 14) (2) “negative and moderate variable mood” (*n* = 204), (3) “positive and moderate variable mood” (*n* = 41), and (4) “negative and highest variable mood” (*n* = 28). The degree of emotional inertia was virtually identical across the profiles.

**Conclusions:**

The real-time monitoring conducted in the present study provides some preliminary indications of different patterns of both average mood and mood variability among MDD patients in treatment in mental health settings. Such varying patterns were not found for emotional inertia.

## Introduction

**Figure 1:**
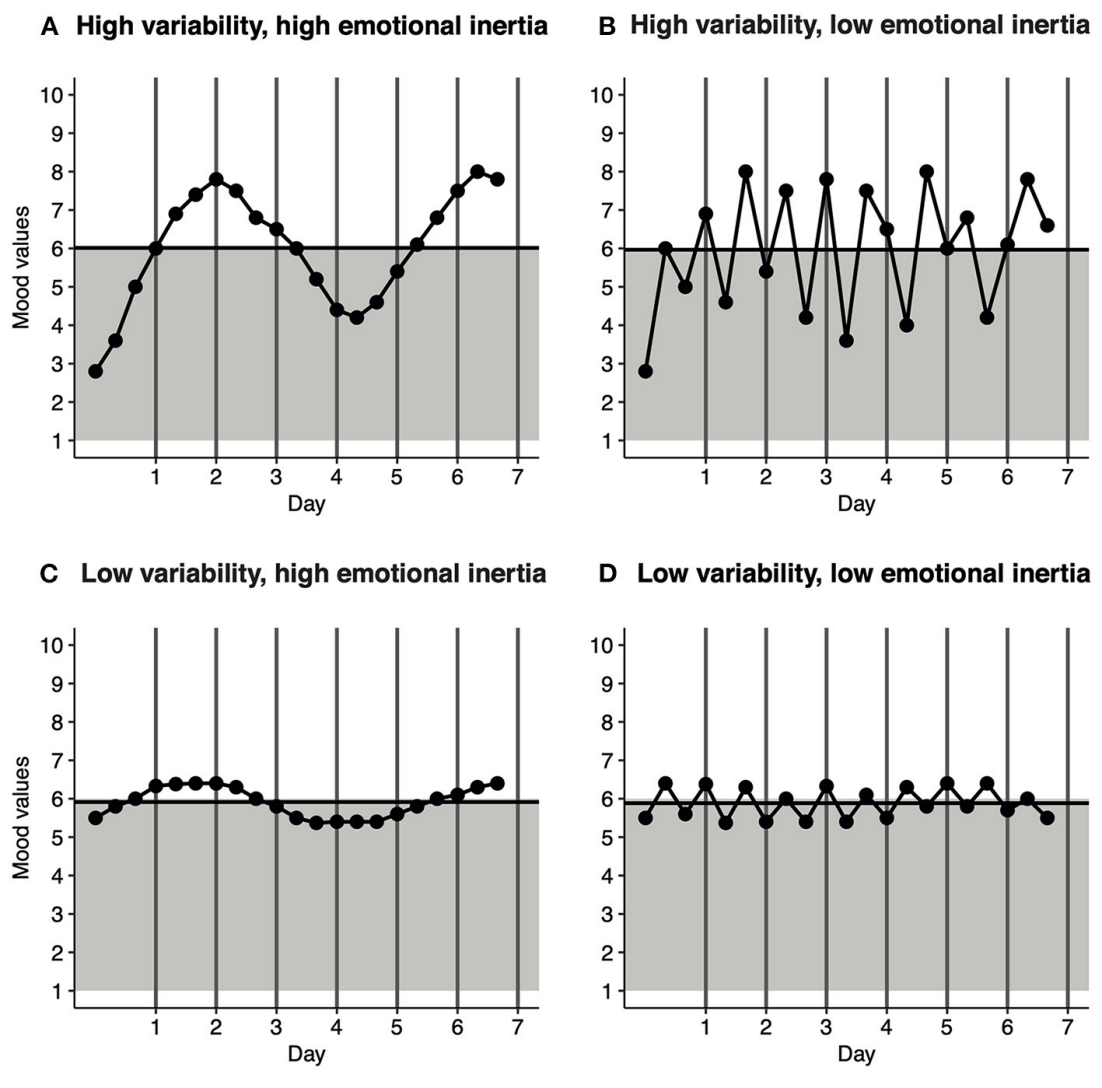
Generated mood dynamic patterns that show different combinations of mood variability and emotional inertia. There were three measurement points over the course of a day. The gray background represents a negative mood (score < 6), while the white area represents a positive mood (≥6). The horizontal black line represents the Average Mood across the 7-day monitoring period. For each panel, the average mood was ~6. **(A)** High variability, high emotional inertia. **(B)** High variability, low emotional inertia. **(C)** Low variability, high emotional inertia. **(D)** Low variability, low emotional inertia.

Even though one of the core symptoms of depression is experiencing “a depressed mood most of the day, nearly every day, for at least 2 weeks”, studies routinely show that the mood of depressed patients is in fact dynamic. It is characterized by highs and lows and is highly responsive to external and internal regulatory processes (1–3). Indeed, there is now a growing body of research that indicates that mood dynamics are an integral part of depression (4–6). In this context, mood dynamics refer to patterns of fluctuations in an individual's mood over the course of a few hours, days or weeks. Here, mood dynamics are designated as a combination of mood variability and emotional inertia (7, 8). Mood variability pertains to the magnitude of a mood shift over a certain period of time (7, 8); that is to say, a patient who displays a larger degree of variability is someone who experiences greater mood shifts during the observed time frame. Emotional inertia refers to the extent to which mood is resistant to change (7, 8); in other words, a patient with a higher level of emotional inertia experiences slower mood shifts. Figure 1 illustrates the various combinations of mood variability and emotional inertia. As one can discern from the images, higher mood variability is suggestive of a larger absolute difference between the peaks and lows (Figures 1A,B), whereas higher emotional inertia is indicative of a mood that tends to linger for a longer time (Figures 1A,C).

Traditionally, mood is assessed by asking retrospective questions (e.g., “How have you felt over the last 2 weeks?”). Indeed, over the years, various retrospective questionnaires and clinical interviews have been found to be reliable and valid for assessing depressive symptoms. However, these types of assessments may not necessarily be suitable for capturing the mood dynamics of depression. For example, most depressed patients experience recall bias (9–11). Consequently, asking a patient to provide a summary of their mood over a prolonged period of time, as when one uses retrospective assessments, can lead to overgeneralizations (10, 12, 13), that are not helpful for capturing fine-grained mood dynamics. To mitigate recall bias, scholars have argued that phenomena should instead be measured as close to their occurrence as possible. For such purposes, ecological momentary assessment (EMA) methods are particularly valuable (14–16). In older forms of research, EMA was either conducted via pen-and-paper diaries or via stand-alone technical devices. Today, EMA data collection is often facilitated by smartphone-based applications (17, 18), which enable patients to provide self-reports on their mental wellbeing or related factors, while in their ecological habitat (19–21). Moreover, the use of pop-up notifications on smartphones allows researchers to randomly select the time point of mood reporting during the day (19–21).

EMA studies have shown that, compared to healthy controls, persons diagnosed with major depressive disorder (MDD) experience, on average, higher variability and higher emotional inertia (4, 22, 23). However, most such studies considered depressed persons to be a homogeneous group. That is problematic, given that other research indicates that mood dynamics in people with MDD are rather heterogenous (24). Exploration of heterogeneity in MDD mood dynamics is therefore of interest, as it provides us with manifold insights, such as into patients' emotional regulation (3, 25). This information may ultimately give rise to different diagnostic and treatment pathways.

We previously conducted an explorative study in a sample of mildly to moderately depressed persons (*n* = 37), who were recruited from the general population (24). We identified two profiles that differed in terms of average mood and mood variability, but not emotional inertia. In addition, we observed that persons who displayed both more negative and variable moods also reported more severe depressive symptoms during baseline testing. This raises the question of whether these profiles are generalizable to a sample of MDD patients seeking treatment. In a meta-analysis by Houben et al. (4), the depressed people in the clinical samples experienced higher levels of variability than those in the non-clinical samples, but no difference in levels of inertia emerged between non-clinical and clinical samples This indicates that a need to explore the possible profiles of mood dynamics in a clinical sample of MDD patients.

To the best of our knowledge, the study we report here is the first that attempts to explore the different profiles of the real-time monitored mood dynamics among MDD patients seeking treatment in routine mental healthcare (MHC). We made use of data on of a subsample of patients (*n* = 287) receiving blended CBT (bCBT) and regular EMA prompts that were collected as part of the trial entitled European Comparative Effectiveness Research on Internet-based Depression Treatment (E-COMPARED) trial (26, 27).

## Materials and Methods

### Participants and Procedures

For the purposes of this study, data were analyzed from a subsample of the E-COMPARED study. E-COMPARED is a randomized controlled non-inferiority trial that was conducted across eight European countries. The primary aim of the trial was to compare the use of bCBT with to treatment-as-usual for people with MDD, with respect to both clinical benefit and cost-effectiveness. The blended treatment combined individual face-to-face (f-t-f) sessions and web- and smartphone-based components into one treatment protocol (28). It included a smartphone-based EMA application that enabled the daily monitoring of mood state, cognitions, social interaction, and sleep patterns. Recruitment for the trial took place from February 2015 to December 2017 in routine MHC settings (26, 27). Patients were recruited from primary care (Germany, Poland, Spain, Sweden and the United Kingdom) and outpatient departments and practices in specialized MHC settings (France, the Netherlands, Switzerland). All patients were asked by their healthcare professional if they were willing to participate. Patients were eligible if they (1) were aged 18 or older; (2) met the DSM-IV criteria for MDD as confirmed by the Mini International Neuropsychiatric Interview (MINI) version 5.0 (29, 30); and (3) reported mild to severe depressive symptoms, scoring ≥ 5 on the Patient Health Questionnaire-9 (PHQ-9) (31). The exclusion criteria were as follow: (1) current receipt of psychological treatment for depression in primary or specialized MHC; (2) current high risk of suicide or a DSM-IV diagnosis of substance dependence, bipolar disorder, psychotic illness or obsessive compulsive disorder as confirmed by the MINI (29, 30); (3) inability to fully comprehend the spoken and written language in their country of residence; (4) no access to a computer with a fast internet connection; (5) no Android-compatible smartphone or unwillingness to use the smartphone provided by the research team. In-depth information about recruitment procedures in countries and settings are provided elsewhere (27).

Patients meeting the inclusion criteria (*n* = 943) were subsequently randomized to either the bCBT (*n* = 476) or treatment-as-usual (*n* = 467) condition. For the purposes of this paper, only patients who were randomized to receive bCBT treatment were initially selected, as the treatment-as-usual group was not invited to take part in the smartphone-based EMA. Of this initial group of 476 patients, 152 did not receive bCBT treatment (that is, never attended the first f-t-f session, dropped out after the first f-t-f session, or never logged onto the platform) or did not provide any EMA data. Of the remaining 324 patients, 37 of the patients had insufficient EMA data (data irretrievable or recorded only once or twice during the first week). This resulted in our final analytic sample of 287 patients.

The studies were conducted in accordance with the Declaration of Helsinki and the rules of Good Clinical Practice. Ethical approval for the trial was obtained locally in each country. All the participants provided written informed consent and granted permission to share their anonymized (encrypted and non-identifiable) data across the participating E-COMPARED partners. Detailed descriptions of both the E-COMPARED study design and the web based-platforms can be found elsewhere (26, 27, 32, 33).

### Measures

#### Patient Characteristics

Information about demographic and clinical characteristics was gathered during baseline testing. Demographic characteristics, which included age, gender and educational level, were collected via an online questionnaire. Clinical characteristics included the severity of the depression, current MDD diagnosis, comorbid psychiatric diagnoses, and the use of medication. The severity of depression was assessed using the online version of the PHQ-9 (31), a brief self-report questionnaire comprising nine items. Each item scores one DSM-IV criterion of MDD on a scale ranging from 0 (not at all) to 3 (nearly every day), as experienced during the preceding 2-week period. The sum of these scores indicates both the presence and severity of depression symptoms, with a minimum score of 0 and a maximum score of 27. Sum scores of 0–4, 5–9, 10–14, 15–19, and 20–27 represented no, mild, moderate, moderately severe and severe depressive symptoms, respectively (31). The PHQ-9 has shown adequate psychometric qualities for detecting depression (31, 34), and good interformat reliability between the pen-and-paper and online versions (35). The MINI, version 5.0 (30), was conducted with the patients by clinicians either over the telephone or f-t-f in order to assess the diagnosis of MDD and of any current comorbid psychiatric disorders. The latter included dysthymia, panic disorder both with or without agoraphobia, agoraphobia, social phobia, generalized anxiety disorder, and post-traumatic stress disorder. Information about medication was gathered using standard questions and included the use of antidepressants, tranquilizers, antipsychotic medication, and sleep medication.

#### Smartphone-Based Ecological Momentary Assessment

As part of the blended treatment, patients were prompted to rate “How is your mood right now?” in the mood-monitoring application on their smartphone. Although different applications were used across the various sites, depending on the local availability of bCBT platforms, the EMA protocol was nonetheless identical across all the sites. The question was answered on a visual analog scale that ranged from 1 (worst) to 10 (best), with one precision digit after the decimal point. To minimize possible treatment effects, only those mood ratings provided during the first seven days of treatment were taken into account. During the first week of treatment, patients only had one f-t-f session to discuss the blended format and were given access to the introductory and psycho-education web-based modules. The EMA monitoring started after the first f-t-f session. During the first seven days of treatment patients were prompted to rate their mood at three separate points of the day: around 10 a.m., 8 p.m. and at a third random time between 10 a.m. and 10 p.m. Although patients were requested to answer the question as quickly as possible, they had a 60-min window to answer. They were also free to answer the question at any other additional time than the fixed prompts. After completion of each questionnaire, both the patient and their therapist were presented with a graph in the application that showed their ratings over time.

### Profile Indicators: Average Mood, Variability, and Emotional Inertia

Average mood (AM) and mood dynamics (variability and emotional inertia) were derived from the mood ratings of the patients over the course of the first week of bCBT treatment, for the reasons noted above. AM refers to the mean scores of the ratings on the EMA questions across the 7-day monitoring period. Although arbitrary, a score above 6 (range 1–10) is typically considered to indicate a positive mood and a score below this cutoff a negative mood (36). Mood variability (MV) pertains to the magnitude of the mood shift and was statistically defined as the standard deviation (SD) of the AM across the 7-day period (7, 8, 37). Although no “official” cutoff values for judging variability have hitherto been established, a larger MV value corresponds to greater mood shifts across the monitoring period. Emotional inertia (EI) refers to the extent to which a person's mood carried over from one assessment (t0) to the next assessment (t1) and is defined statistically as the autocorrelation (37). The value of EI theoretically ranges from−1 to 1. A negative EI indicates that if the score at t-1 was above the AM value, then the score at t is more likely to be below the AM value, and vice versa (38). The larger a negative EI over time, the lower the level of emotional inertia (that is, faster mood shifts occur across the monitoring period) (4, 8). Positive EI mean that a higher or lower score at t-1 corresponds to a higher or lower score at t (38). A larger positive EI over time thus equals higher emotional inertia (with slower mood shifts across the monitoring period) (4, 8). As yet there are no established cutoff values for when EI is considered to be low, moderate, or high.

### Statistical Analyses

**Figure 2:**
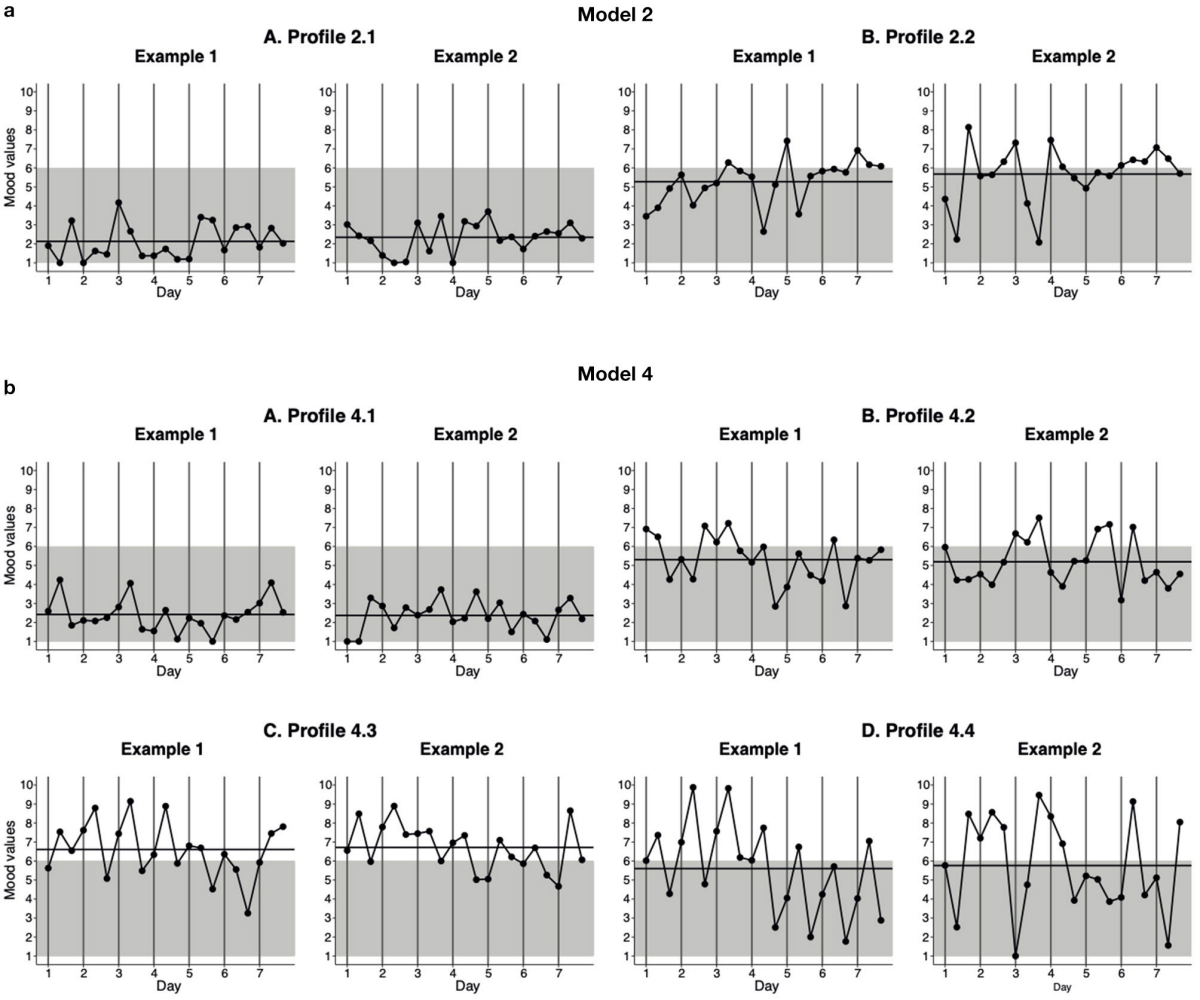
Generated EMA-response patterns of the profiles of the two- and four-profile models. The data for these graphs were generated using the standardized mean scores for the indicators of the profiles using the estimated parameters shown in **Table 2**. Three EMA assessments were completed each day, at 10 a.m., 8 p.m., and at a random time between 10 a.m. and 10 p.m. The horizontal black line represents the AM across the 7-day monitoring period, while the gray background represents negative mood (score < 6) and the white area represents positive mood (≥6). **(a)** Model 2. **(b)** Model 4.

We conducted Latent profile analyses (LPA), a type of cluster analysis that can classify patients into profiles based upon a set of continuous variables (39). LPA enables searching for a model with the “optimum” number of profiles. To choose the optimum number of profiles, we first considered multiple fit indices (39) and then evaluated the clinical relevance of the profiles. The indices included the Bayesian information criterion (BIC) (40) and the Akaike information criterion (AIC) (41), with lower BIC and AIC values indicating better model fit (39–41), under the assumption that a difference of delta < 2 between the values of two different models was negligible (42, 43). We also considered the bootstrapped likelihood ratio test (BLRT) and Lo-Mendell-Rubin adjusted likelihood ratio test (LMRA-LRT) (44) were also considered. These assess whether a model with K profiles fits the data better than a model with K−1 profiles, whereby a significant *p*-value supports the more complex model (44, 45). We then evaluated the classification accuracy of the models that potentially best fitted the data and we compared the prevalence rates of the profiles. Classification accuracy was evaluated in terms of the entropy value of each model (46) and the mean posterior probabilities of each profile within the models (47). Both such values theoretically range from 0 to 1 (46, 47). Although no official cutoff values available, an entropy value of ≥0.80 and mean posterior probability values of ≥0.70 for every model within the model are generally considered as being indicative of an adequate classification (46, 47). The clinical relevance of the models was evaluated by examining the standardized mean scores for the indicators. After deciding on the best fitting model, we assigned patients to their most likely profile (based on higher posterior probability) before calculating descriptive statistics of the demographic and clinical characteristics. Pearson's chi-square tests, Kruskall-Wallis and Mann-Whitney pairwise comparisons were performed to examine interprofile differences. In order to check for any selection bias, we performed a comparative analysis of the baseline demographic and clinical characteristics of the patients in the analytic sample (*n* = 287) vs. the E-COMPARED bCBT patients that did not meet this study's inclusion criteria (*n* = 189). A *p*-value of smaller than <0.05 was considered statistically significant. To counteract the problem of multiple testing of the pairwise-comparisons, we applied Holm-Bonferroni sequential corrections (48). All our analyses were performed in RStudio (R Version 4.0.2.). The TidyLPA R package was used for the LPA (49) and the emaph R package (37, 50) was used to generate data for the visual presentations included in Figures 1, 2.

## Results

### Characteristics of the Study Sample

The sample comprised 287 participants, of whom 67% (*n* = 191) were female. The mean age was 39.4 years (SD = 13.7); 43% (*n* = 123) had elementary or secondary education and 57% (*n* = 164) had higher education. All patients were diagnosed with MDD as primary diagnosis and reported moderately severe depressive symptoms on average (PHQ-9; M = 15.5, SD = 4.8). One or more comorbid diagnoses were reported by 59% of the patients (*n* = 169). Finally, 32% (*n* = 92) of the patients were currently using antidepressant medication.

The analyses that were conducted to examine potential selection bias showed that the E-COMPARED patients who were allocated to receive bCBT treatment but did not meet this study's inclusion criteria (*n* = 476 – 287 = 189) did not differ in terms of their baseline demographic and clinical characteristics from those patients who were included in our study (*n* = 287).

### Latent Profile Analyses: Choosing the Best Fitting Model

Table 1 provides the fit indices of the models along with the different number of profiles estimated. The results were limited to five-profile models, because the software indicated that models with more than five profiles were too complex for estimations using these data. The BIC value was in favor of the two-profile model, followed by the four-profile model. The AIC preferred the four-profile model, followed by the five-profile model. The BLRT supported both the two- and four-profile solutions, while the LMRA-LRT posited that increasing to five profiles would yield a better model. In summary, the two-profile solution was supported by BIC and BLRT, while the four-profile solution was supported by AIC and BLRT, as well as being the second-best solution according to the BIC values; the LMRA-LRT supported the more complex models. Based on these five different fit indices, model 2 and model 4 were deemed to both potentially fit the data.

**Table 1:** Fit indices of the latent profile analyses.

**Model**	**LLH**	**AIC**	**BIC**	**BLRT**	**LMRA-LRT**	**Entropy values**	**Mean posterior probabilities**				
				***p***	***p***		**1**	**2**	**3**	**4**	**5**
Two	−773.85	1,567.70	1,604.30	0.01	<0.001	0.86	0.83	0.97			
Three	−770.98	1,569.95	1,621.19	0.25	<0.001	0.78	0.84	0.93	0.71		
Four	−759.58	1,555.17	1,621.04	0.01	<0.001	0.67	0.85	0.84	0.73	0.76	
Five	−757.85	1,559.70	1,640.21	0.59	<0.001	0.71	0.80	0.85	0.78	0.68	0.80

*LLH, loglikelihood; AIC, Akaike information criterion; BIC, Bayesian information criterion; BLRT, Bootstrapped likelihood ratio test; LMRA-LRT, Lo-Mendell-Rubin adjusted likelihood ratio test*.

We next evaluated the classification accuracy (entropy values and mean posterior probabilities) of models 2 and 4 (Table 1). For model 2, the entropy value (≥0.80) and mean posterior probabilities (≥0.70 for both profiles) indicated an acceptable classification. Although model 4 did not meet the cutoff of 0.80 for the entropy value, the mean posterior probabilities of the profiles did indicate an acceptable classification (≥0.70 for all four profiles). Since the cutoffs were somewhat arbitrary and the mean posterior probabilities supported model 4, that model was not discarded.

Finally, we evaluated the clinical relevance of the models by first examining the standardized mean scores of models 2 and 4 separately and then comparing the two models. See Table 2 for the standardized mean scores for each of the indicators in the two models and Figure 2 for the generated EMA response patterns of the profiles of the two models. First, the examination of model 2 (Figure 2a) revealed that the two profiles differed in terms of average mood (AM) and mood variability (MV), but not in terms of emotional inertia (EI). The patients assigned to profile 2.1 (Figure 2a, A, prevalence 5%) were characterized by a more negative and less variable mood (AM = 2.88, MV = 0.74) than the patients in profile 2.2 (Figure 2a, B, prevalence 95%), who experienced a rather more positive and more variable mood (AM = 5.50, MV = 1.38). Second, the examination of model 4 (Figure 2b) revealed four profiles that differed in terms of average mood and variability of mood, but not with respect to emotional inertia. The patients belonging to profile 4.1 (Figure 2b, A, prevalence 5%) experienced the most negative and least variable mood (AM = 2.53, MV = 0.67), in comparison to the patients in the other three profiles. The patients in profile 4.2 (Figure 2b, B, prevalence 71%) were characterized by a more positive and more variable mood (AM = 5.11, MV = 1.24) than those in profile 4.1. The patients in profile 4.3 (Figure 2b, C, prevalence 14%) experienced a positive mood, but displayed similar variability to those in profile 4.2 (AM = 6.68, MV = 1.32). Finally, although the patients in profile 4.4 (Figure 2b, D, prevalence 10%) experienced similar moods on average to those patients in profile 4.2, they experienced the highest level of mood variability (AM = 5.61, MV = 2.38) in comparison with the patients in the other three profiles.

**Table 2:** Standardized mean scores for each of the indicators in the two- and four-profile models.

	**Prevalence**	**Average mood**	**Mood variability**	**Emotional inertia**
		**M (SE)**	**M (SE)**	**M (SE)**
**Model 2**
Profile 2.1	5%	2.88 (0.58)	0.74 (0.14)	−0.01 (0.07)
Profile 2.2	95%	5.50 (0.11)	1.38 (0.05)	0.03 (0.02)
**Model 4**
Profile 4.1	5%	2.53 (0.51)	0.67 (0.16)	−0.02 (0.08)
Profile 4.2	71%	5.11 (0.25)	1.24 (0.08)	0.04 (0.03)
Profile 4.3	14%	6.68 (0.50)	1.32 (0.22)	−0.00 (0.17)
Profile 4.4	10%	5.61 (0.16)	2.38 (0.19)	0.02 (0.15)

*Calculated over the 7-day monitoring period. SE, standard error*.

We consequently reached the conclusion, in the comparison of model 2 with model 4, that profile 2.1 and profile 4.1 displayed similar patterns. However, while in model 2 all the remaining patients were assigned to a single profile (2.2), the remaining patients in model 4, the remaining patients were distributed across three models (4.2, 4.3, and 4.4). We therefore argue that model 4 is more clinically relevant, providing us with four conceptually meaningful profiles. Choosing model 2 would risk omitting some of the meaningful differences among MDD patients in terms of mood dynamics. We therefore deemed model 4 to be the best fitting model.

### The Best Fitting Model: Demographic and Clinical Characteristics for Each Profile

Demographic and clinical characteristics for both the full sample and each of the four profiles in the best fitting model are presented in Table 3. In the baseline testing, statistically significant differences emerged between the four profiles in terms of severity of depressive symptoms (*p* = 0.01) as measured by the PHQ-9. Subsequently, Holm-Bonferroni-adjusted pairwise comparisons showed that the patients belonging to profile 4.3 experienced significantly fewer depressive symptoms than the patients in both profile 4.1 (U = 438.0, *p* = 0.009), profile 4.2 (U = 5,403.5, *p* = 0.009), and profile 4.4 (U = 386.0, *p* = 0.022). No other significant pairwise-comparison differences were found in terms of depression severity at baseline. The statistical tests likewise revealed no differences between the four profiles with respect to demographic characteristics, number of completed EMA assessments, presence or absence of co-morbid DSM-IV diagnoses, and antidepressant use (yes/no).

**Table 3:** Descriptive statistics for the demographic and clinical characteristics of the best fitting model.

	**Total**	**Profile 4.1**	**Profile 4.2**	**Profile 4.3**	**Profile 4.4**	**Test value**	***p*-value**
*n*	287	14	204	41	28		
**Demographics**							
Age	39.4 (13.7)	39.0 (12.1)	39.6 (13.5)	40.6 (15.9)	36.6 (14.2)	H(3) = 1.35	0.72
Female	191 (67%)	9 (64%)	136 (67%)	25 (61%)	21 (75%)	χ^2^(3) = 4.45	0.68
Educational level							
Elementary or secondary	123 (43%)	5 (36%)	87 (43%)	18 (44%)	13 (46%)	χ^2^(3) = 0.46	0.93
Higher	164 (57%)	9 (64%)	117 (57%)	23 (56%)	15 (54%)		
Completed EMA	11.7 (8.0)	12.4 (13.7)	11.46 (7.0)	13.0 (10.5)	10.7 (7.2)	H(3) = 1.27	0.74
**Clinical characteristics**							
PHQ-9 at baseline	15.5 (4.8)	17.9 (5.4)	15.7 (4.6)	13.1 (4.8)	16.3 (5.5)	H(3) = 12.70	0.01
Co-morbid DSM-IV diagnoses^*a*^							
Yes	169 (59%)	9 (64%)	123 (60%)	23 (56%)	14 (50%)	χ^2^(3) = 1.25	0.74
Antidepressant use							
Yes	92 (32%)	8 (57%)	66 (32%)	11 (27%)	7 (25%)	χ^2^(3) = 5.21	0.16

*Pearson's chi-square tests and Kruskall-Wallis tests were used as appropriate. a: “Yes” indicates that the patient experienced at least one comorbid diagnosis. Comorbid diagnoses included dysthymia, panic disorder with agoraphobia, panic disorder without agoraphobia, agoraphobia, generalized anxiety disorder, and post-traumatic stress disorder*.

## Discussion

The results of this explorative study in a cross-national sample of MDD patients demonstrated variations in average mood and mood variability, but not in emotional inertia. Assessments were based on data derived from smartphone-based EMA during the first week of bCBT. The analyses revealed four profiles, which we labeled as “very negative and least variable mood”, “negative and moderate variable mood”, “positive and moderate variable mood”, and “negative and high variable mood”. The majority of patients fitted the profile “negative and moderate variable mood”.

In agreement with our previous explorative study (24), in which profiles of mood dynamics among a non-clinical sample of depressed persons, profiles emerged here differed in terms of average mood and mood variability, but not in terms of emotional inertia. The fact that we did not find interprofile differences for emotional inertia was in line with previous research, which also did not associate emotional inertia with the level of depressed mood (4, 24). However, the characteristics of average mood and mood variability in the identified profiles did differ markedly between our two studies. While our previous study negative mood was associated with greater variability, the patients in the present study who experienced a more negative mood displayed the lowest level of mood variability. At first glance, these results might sound somewhat surprising, in that they contrast with the meta-analysis conducted by Houben et al. (4). Their meta-analysis, which compared sample groups (non-clinical and clinical samples) with different levels of depression, showed that higher levels of depression severity were associated with higher levels of mood variability.

Although more speculative and not investigated in this paper, two of the four profiles identified in the present study could potentially be linked to already well-grounded subtypes of depression, as they show similar characteristic features. The first profile (“very negative and least variable mood”) shows similarities with depression with melancholic features, or melancholic depression as it is otherwise known. One characteristic feature of melancholic depression is a persistent negative mood combined with a lack of mood reactivity, which means that even when something good happens, a person's mood does not brighten (51, 52). Although we did not investigate mood reactivity, it indeed appears that the persons in this profile experienced little brightening in their mood; they displayed a very negative mood with little variability across the monitoring period. The patients in the second profile (“negative and moderate variable mood”) resembled patients experiencing depression with atypical features, or atypical depression. Although the term “atypical” itself is ordinarily associated with rarity, atypical depression is certainly not rare in terms of depressive disorders (53, 54). In contrast to patients with melancholic depression, patients diagnosed with atypical depression do show a degree of mood reactivity (51, 54, 55). Although, as noted, we are not in a position to make any firm statements about mood reactivity, the patients in the second profile did indeed appear to experience some brightening of their mood, in that they displayed variable moods across the monitoring period. Whether or not such profiles of mood dynamics may indeed be linked to melancholic or atypical depression should be investigated in future research.

The two other profiles, “positive moderate variable mood” and “negative and highest variable mood” are more difficult to explain within the framework of the classical clinical features of MDD. First, identifying a pattern labeled “positive moderate variable mood” in a sample of MDD patients seeking treatment appears to be counterintuitive. One explanation is that the patients who were assigned to this profile might have experienced a spontaneous decrease in their depression symptoms (56). In primary care settings, 23% of people with untreated depression will remit within a 3-month period (56). In our study, although patients were diagnosed with MDD during their therapy intake assessment, some did not actually begin treatment until later due to long waiting lists. Another potential reason for the positive mood state during the monitoring period could be that the patient intake process itself, that is, the initial f-t-f contact with a healthcare professional, could have caused some patients' mood to brighten. A second explanatory difficulty arises from the high mood variability among patients in the fourth profile, labeled “negative and highest variable mood”, which is an especially notable finding. Patients assigned to this profile experienced similar moods on average to the patients in the second profile, which showed similarities with atypical depression. However, patients in the fourth profile experienced notably large mood shifts, perhaps too large to fit the classical picture of atypical depression. Their mood pattern was more reminiscent of those exhibited by patients with conditions like borderline personality disorder, bipolar depression, or attention deficit hyperactivity disorder, which are all characterized by considerable mood instability (51). It can be hard to distinguish these disorders from MDD, as depressive symptoms are part of their respective psychopathology (57–59). On the other hand, distinct unipolar MDD does also often co-occur with borderline personality disorder and attention deficit hyperactivity disorder (57–59). What needs future exploration is therefore whether or not the two mood dynamics profiles in question are indeed reflective of patients experiencing a spontaneous decrease of depression symptoms or of patients with a potential other, possibly comorbid, psychiatric diagnosis.

The results of this explorative study should be considered with certain limitations in mind. First, seeking to generalize the results of LPA studies to broader settings should always be done with caution. Generally speaking, drawing direct comparisons between an LPA study and the findings of other studies is often difficult in light of differences in the sample groups, sampling protocols and statistical methods used (60). An additional issue is that the use of LPA techniques to analyze EMA data is a novel approach and is still very much in its experimental phase. Indeed, there is only one other study in this area, at least to our knowledge, that has successfully applied LPA to EMA data (61), besides our previous study described above (24). The former study applied LPA to smartphone-based EMA data of suicidal thoughts and revealed distinctive profiles of suicidal thinking that differed in the intensity and the variability of suicidal thoughts (61). A second limitation lies in the lack of available psychometric qualities of our EMA measure of mood (36, 62). Investigating psychometric qualities of various EMA measures of mood is an important issue, yet in need of more research (63). Fortunately, available results are potentially positive. For example, a recent exploratory study found that clinician ratings on the 6-Item version of the Hamilton Depression Rating Scale (HAM-D_6_) were strongly correlated with the EMA HAM-D_6_ (64). Third, there is a lack of validated cutoff scores for average mood, mood variability, and emotional inertia. As a result, we were only able to describe the differences in the scores, as opposed to interpreting the absolute values. Fourth, the limited number of assessments for each patient should also be noted. That derives from the fact that the adherence rates in this study were lower than those in previous EMA studies among persons with MDD. For example, two meta-analyses examined adherence rates in EMA studies among persons with MDD, reporting rates ranging from 65 to 90% across the included studies (17, 65). However, those adherence rates may not be generalizable to our study, for the simple fact that most such studies were carried out in a research context, rather than in a routine MHC setting. Fortunately, Houben et al.'s (4) meta-analysis did demonstrate that mood dynamics display a certain self-similarity across different time frames. They included studies with a minimum of three measures, with a maximum period of 1 week in between consecutive measurements, while the time frames did not moderate the relation between mood dynamics and psychological wellbeing. Moreover, in our study the adherence rates did not differ across the identified profiles. A fifth limitation in our study is that the entropy value of our best fitting model was not particularly high. However, the mean posterior probabilities for the most suitable profiles did indicate acceptable classification quality. Sixth, the timing of the monitoring period should also be considered when drawing conclusions from the present study. As described in the introduction, we opted to consider only the first week of treatment so as to minimize possible treatment effects. However, starting treatment can itself cause mood shifts, and that could in turn alter the potentially identifiable profiles. A final point to consider is the external validity of our findings. Fortunately, our sample comprised patients being treated for depression across various settings in eight European countries; nonetheless, external validity remains a key concern in every study with a focus on depression. As a result of this series limitations of, we should be very cautious when generalizing our results to broader settings.

Nevertheless, we believe our study has specific implications for future clinical practice and future studies. Mood monitoring is part of the CBT and bCBT protocols for depression, because mood is seldom completely stagnant (1–3), even though a “persistent negative mood” is defined as one of the core symptoms of depression (1–3). Mood monitoring thus serves as a starting point for a range of other behavioral activation techniques (66, 67). Indeed, previous studies have already underscored the potential for other EMA methods to be utilized as an add-on tool in treating depression (68–70). Our study augments this potential by suggesting that a smartphone-based EMA application can be a helpful tool for identifying and monitoring variations in mood dynamics among MDD patients. It could provide patients and their therapists with insights in the patterns of mood (and related factors) and make clearer to them into which profile of mood dynamics the patient fits. Such profile information may also provide therapists with interesting diagnostic information and suggest different treatment pathways, as well as helping to identify psychiatric comorbidity in early stages of treatment. At this juncture, however, further research is needed to provide more robust results before the findings have direct clinical implications. Studies in larger samples and varied settings are needed in order to increase the external validity of our findings. Also, it would be worthwhile to apply machine learning based methods in future studies. For example, artificial intelligence techniques that extract latent variables from a full dataset using autoencoders and subsequently cluster these latent variables. We could compare these findings with our findings and see whether or not the identified profiles of mood dynamics depend on the statistical approach.

In conclusion, this study identified four profiles based on average mood and mood variability among MDD patients, which were measured using smartphone-based EMA during the first week of bCBT. No distinctive patterns emerged for emotional inertia. Despite some of the acknowledged limitations, the results provide indications for the existence of several different patterns of mood dynamics in MDD, as revaled by real-time monitored average mood and mood variability.

## Data Availability Statement

The data that support the findings of this study are available upon reasonable request from the authors. Requests to access these datasets should be directed to E-COMPARED, info@e-compared.eu.

## Ethics Statement

The studies involving human participants were reviewed and approved by the Local Ethics Committee in each country. The patients/participants provided their written informed consent to participate in this study.

## Author Contributions

CG, JSc, AH, JSm, and HR: conceptualization. CG and AH: methodology and formal analysis. RA, GA, RB, TB, CB, AC, RC, DE, AG-P, J-BH, RH, JH, LK, AK, TK, AR, IT, and NT: resources. CG: writing—original draft and visualization. JSc, AH, RA, GA, RB, TB, CB, AC, RC, DE, AG-P, J-BH, RH, JH, LK, AK, TK, AR, IT, NT, JSm, and HR: writing—review and editing. JSc, AH, JSm, and HR: supervision. All authors read and approved the submission of the final manuscript.

## Funding

The European COMPARative Effectiveness research on blended depression treatment versus treatment-as-usual (E-COMPARED) project, was funded by the European Commission FP7-Health-2013-Innovation-1 program, Grant Agreement Number: 603098-2.

## Conflict of Interest

DE has served as a consultant to/on the scientific advisory boards of Sanofi, Novartis, Minddistrict, Lantern, Schoen Klinike, Ideamed and German health insurance companies BARMER, Techniker Krankenkasse and a number of federal chambers for psychotherapy. He is also stakeholder of the Institute for health training online formerly GET.ON/ now HelloBetter, which aims to implement scientific findings related to digital health interventions into routine care. IT reports to have received fees for lectures/workshops in the e-mental-health context from training institutes for psychotherapists. The remaining authors declare that the research was conducted in the absence of any commercial or financial relationships that could be construed as a potential conflict of interest.

## Publisher's Note

All claims expressed in this article are solely those of the authors and do not necessarily represent those of their affiliated organizations, or those of the publisher, the editors and the reviewers. Any product that may be evaluated in this article, or claim that may be made by its manufacturer, is not guaranteed or endorsed by the publisher.

## Abbreviations

AC: Autocorrelation
AM: Average mood
AIC: Akaike information criterion
APA: American Psychiatric Association
bCBT: Blended Cognitive Behavioral Therapy
BIC: Bayesian information criterion
BLRT: Bootstrapped likelihood ratio test
E-COMPARED: European COMPARative Effectiveness trial
EI: Emotional inertia
EMA: Ecological momentary assessment
F-t-f: Face-to-face
IQR: Interquartile range
LMRA–LRT: Lo–Mendell–Rubin adjusted likelihood ratio test
LPA: Latent profile analysis
MDD: Major depressive disorder
MHC: Mental healthcare
PHQ-9: Patient Health Questionnaire-9
SE: Standard error
MV: Mood variability.
